# Compact tape-driven sample delivery system for serial femtosecond crystallography

**DOI:** 10.1107/S1600576726000063

**Published:** 2026-02-09

**Authors:** Jungmin Kang, Yoshiaki Shimazu, Fangjia Luo, Ayumi Yamashita, Tomoyuki Tanaka, Yuichi Inubushi, Kensuke Tono, Nipawan Nuemket, Allen M. Orville, So Iwata, Eriko Nango, Makina Yabashi

**Affiliations:** a RIKEN SPring-8 Center, 1-1-1 Kouto, Sayo-cho, Sayo-gun, Hyogo679-5148, Japan; b Japan Synchrotron Radiation Research Institute 1-1-1 Kouto, Sayo-cho Sayo-gun Hyogo679-5198 Japan; c Diamond Light Source Harwell Science and Innovation Campus Didcot OxfordshireOX11 0DE United Kingdom; d Research Complex at Harwell Harwell Science and Innovation Campus Didcot OxfordshireOX11 0FA United Kingdom; e Kyoto University Yoshida-konoe-cho, Sakyo-ku Kyoto606-8501 Japan; f Tohoku University 2-1-1 Katahira, Aoba-ku Sendai980-8577 Japan; SLAC National Accelerator Laboratory, Menlo Park, USA

**Keywords:** serial femtosecond crystallography, X-ray free-electron lasers, room-temperature crystallography, conveyor belts, drop-on-tape sample delivery, nanolitre droplets

## Abstract

We have developed a compact tape drive (CoT) with on-demand sample delivery for time-resolved serial femtosecond crystallography (SFX) experiments, which can deliver sample droplets and/or initiate reactions with a drop-on-drop strategy. Two disposable piezoelectric injectors are positioned in tandem along the tape to produce a queue of nanolitre-scale droplets. X-ray free-electron laser pulses arrive perpendicular to and pass through the broad face of the tape. The pulse is synchronized and aligned to the droplets, thereby enabling highly efficient SFX data collection. The tape transport speed and the delivery distance can be varied to control the mixing time from approximately 130 ms to tens of seconds. We conducted time-resolved SFX experiments utilizing a basic enzymatic reaction model of hen egg white lysozyme (HEWL) and *N*-acetyl-*D*-glucosamine (GlcNAc) to demonstrate the drop-on-drop capabilities of the CoT, and the full binding process of GlcNAc to HEWL was observed at 1.3–9.7 s.

## Abstract 2

A tape-driven liquid sample droplet delivery system for serial femtosecond crystallography is described.

## 1. Introduction

Serial femtosecond crystallography (SFX) is a method to determine crystal structures using an X-ray free-electron laser (XFEL) (Chapman *et al.*, 2011 ▸; Barends *et al.*, 2022 ▸). In SFX, diffraction patterns are collected from randomly oriented microcrystals under room-temperature conditions with intense femtosecond X-ray pulses before the onset of radiation damage (Neutze *et al.*, 2000 ▸). When combined with reactions triggered by light, mixing with a substrate and/or heating, these diffraction patterns measured at near-physiological temperatures allow for the real-time observation of the structural dynamics and chemical reactions that occur in protein crystals. Time-resolved SFX (TR-SFX), which combines a reaction initiator and SFX, is widely used to visualize the structural changes and reactions in proteins (Tenboer *et al.*, 2014 ▸).

SFX requires pristine microcrystals to be supplied to the XFEL intersecting area for each pulse because the crystals are damaged after irradiation. Thus, various sample delivery methods have been employed to supply crystals to the XFEL intersecting area. These can be classified into two main types: continuous flows and discrete droplets. The continuous type, such as a liquid jet or high-viscosity sample injector, carries crystals suspended in a buffer or embedded in a high-viscosity carrier. A typical SFX experiment requires a crystal slurry with a high crystal density (crystals per millilitre) to achieve an optimal hit rate (*i.e.* percentage of XFEL pulses diffracted by crystals) of 20–60%. Thus, crystals are wasted during the interval between XFEL pulses, which increases sample consumption, especially at low repetition rates of several tens of hertz. The discrete type includes our previously reported pulsed liquid droplet injector (Mafuné *et al.*, 2016 ▸), which uses a droplet nozzle with an inner diameter of 80 µm piezo-driven in synchronization with an XFEL pulse to eject around 0.3 nl droplets containing microcrystals, and acoustic droplet ejection (ADE) (Roessler *et al.*, 2016 ▸), which uses focused sound waves to eject a 2–3 nl volume droplet into an XFEL pulse. Discrete-type sample delivery methods do not waste crystals during the interval between XFEL pulses and thus reduce sample consumption. However, ensuring that the XFEL pulses consistently hit crystals in micrometre-scale droplets is a major challenge, especially if the crystals are heterogeneous in size, which affects the droplet ejection speed and stability. In addition, droplets move at approximately 14 m s^−1^, and their trajectory wanders more with longer travel distances, both of which limit the delay time for TR-SFX experiments to around 2 µs (Kubo *et al.*, 2017 ▸).

To address these challenges, Fuller *et al.* (2017 ▸) introduced the drop-on-tape (DOT) method, which utilizes a tape as a conveyor belt to form a sequence of droplets and ensure their precise arrival at the XFEL intersecting area. Their setup uses ADE (Roessler *et al.*, 2016 ▸) to eject 0.8–6.0 nl droplets synchronized to XFEL pulses onto the tape, which is driven at a speed of 30–600 mm s^−1^. The tape surface is positioned parallel to the XFEL beam to enable simultaneous SFX and X-ray emission spectroscopy from each drop and X-ray pulse. After irradiation, the tape is cleaned and reused for sub­sequent droplets. To demonstrate the capabilities of the DOT method, Fuller *et al.* conducted pump–probe experiments using light excitation with photosystem II and time-resolved experiments involving the gas activation of ribonucleotide reductase R2. They also conducted time-resolved mixing experiments by combining the DOT method with an additional piezoelectric injector that dispensed picolitre-scale ligand droplets onto a nanolitre-scale sample droplet to induce turbulence for mixing as the droplets merged (Butryn *et al.*, 2021 ▸). Butryn *et al.* used hen egg white lysozyme (HEWL) and *N*-acetyl-*D*-glucosamine (GlcNAc) and were able to visualize the process of GlcNAc binding to active sites over time, whereas Nguyen *et al.* (2020 ▸) used CYP121 (a P450 enzyme from *Mycobacterium tuberculosis*) and peracetic acid binding to initiate a peroxide shunt reaction.

Here, we report a compact tape-driven sample delivery system (CoT) for mix-and-inject SFX experiments based on the DOT method. Instead of positioning the tape surface parallel to the XFEL beam, the CoT approach positions the tape so that so that the pulses penetrate a droplet on the tape vertically, to facilitate alignment between the XFEL intersecting area and the sample droplets. While the DOT method cleans and dries the tape *in situ* for continuous use over extended periods of time, the CoT utilizes a reel-to-reel tape drive and single-use tapes. Piezoelectric injectors with disposable nozzles are used to eject sample droplets. To demonstrate the capabilities of the CoT, we conducted time-resolved mixing SFX experiments at the SPring-8 Angstrom Compact Free-Electron Laser (SACLA) facility using HEWL crystals and GlcNAc.

## 2. Compact tape-driven sample delivery setup

**Figure 1:**
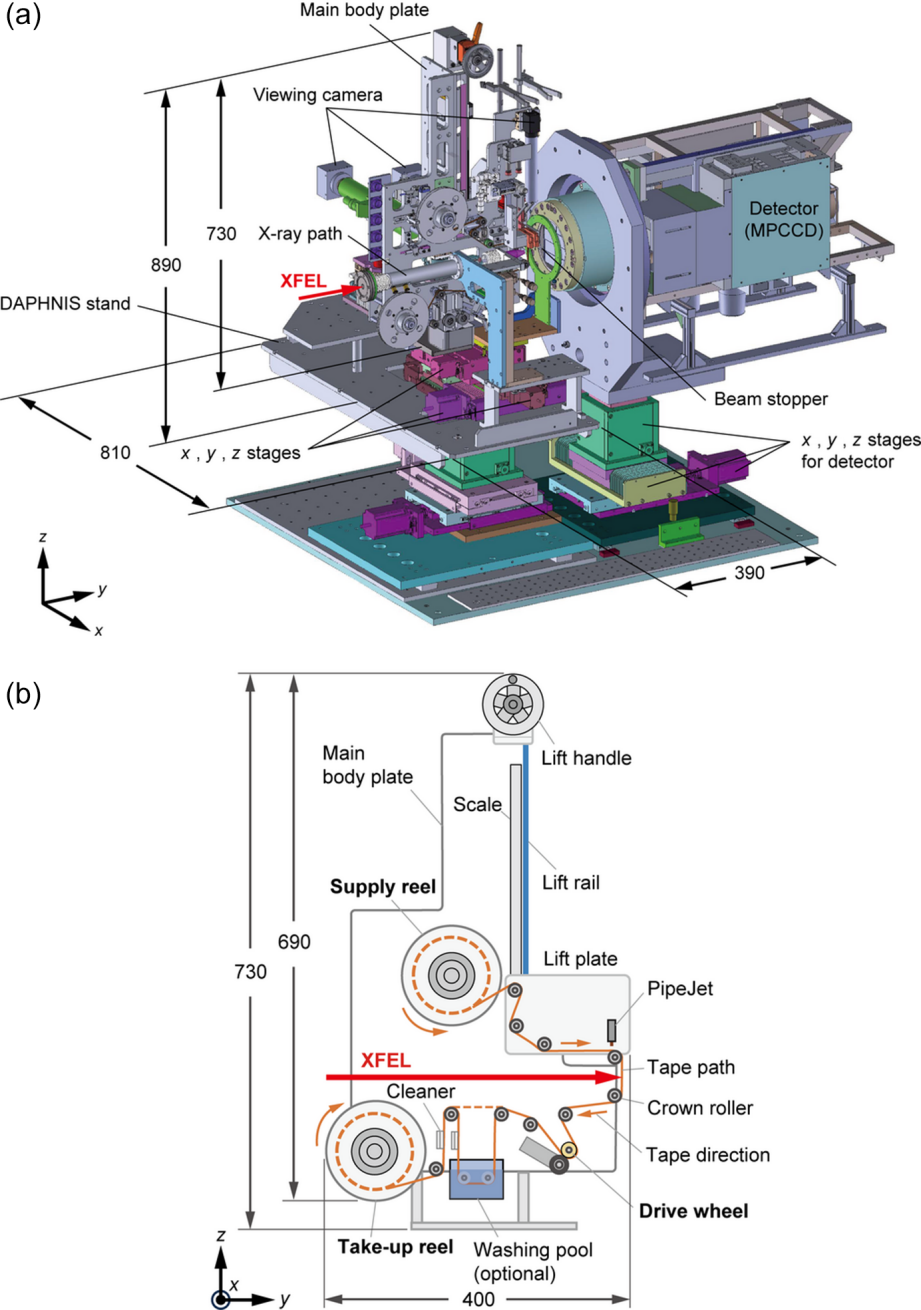
(*a*) Computer-aided design diagram of the CoT installed on DAPHNIS. (*b*) Schematic diagram of the main body plate. Units are in millimetres.

The CoT comprises a drive wheel, tape reels, crown rollers, disposable piezoelectric injectors with sample supply components, a lift unit for changing the distance between the samples and the X-ray interaction point, and stepping motors, which are all mounted on a 12 mm-thick duralumin plate. Fig. 1 ▸(*a*) shows the CoT mounted on DAPHNIS (diverse application platform for hard X-ray diffraction in SACLA) (Tono *et al.*, 2015 ▸), which is equipped with a multiport charge-coupled device (MPCCD) detector (Kameshima *et al.*, 2014 ▸). The main body is 400 mm wide and 730 mm high, and the *x*-, *y*- and *z*-axis stages for aligning the sample droplets with the XFEL intersecting area are installed below the body. A collimator through which XFEL beams pass is fixed to the DAPHNIS stand and is separated from the main body plate. Helium gas flows inside the collimator to prevent parasitic scattering, while the entire setup is kept under atmospheric conditions. Cameras are used to monitor the ejection of the sample droplets and their delivery to the XFEL intersecting area.

This device is also designed to enable pump–probe TR-SFX experiments. An optical system for photoexcitation of crystals on the tape can be introduced into the setup by delivering a nanosecond laser beam, such as an optical parametric oscillator (OPO) or the second-harmonic generation (SHG) output of an Nd:YAG laser, in a direction perpendicular to the XFEL beam (Kubo *et al.*, 2017 ▸).

Fig. 1 ▸(*b*) shows a schematic diagram of the CoT. A washing pool can be introduced if needed. The reel-to-reel tape drive, which is widely used in serial crystallography at synchrotron facilities (Beyerlein *et al.*, 2017 ▸; Zielinski *et al.*, 2022 ▸; Henkel *et al.*, 2023 ▸), comprises a 100 m-long roll of polyimide film (Kapton) tape without any water-repellent treatment on the surface, with a thickness of 12.5 µm and width of 3 mm, that is inserted into a reel cartridge of two 130 mm-diameter discs [Fig. S1(*a*) in the supporting information]. The reel cartridge is set on the supply reel unit and rotates counterclockwise to release the tape. The drive wheel comprises a stainless steel wheel with a diameter of 20 mm and width of 20 mm that is coated in a urethane rubber layer with a thickness of 3 mm. It is placed at the bottom and rotates clockwise to convey the tape at speeds of 15–300 mm s^−1^. It is possible to set the tape speed to less than 15 mm s^−1^ (down to approximately 15 µm s^−1^ for the currently used stepper motor), but droplets dispensed onto the tape without any water-repellent treatment may not separate. Here, the pulse motor connected to the supply reel applies a weak force in a clockwise direction, which is opposite to the direction of the supply reel’s rotation, to tension the tape. A take-up reel unit with an empty reel cartridge also rotates clockwise to collect the tape at the end of the tape path. Ten crown rollers with a diameter of 16 mm, a width of 7.4 mm and a curvature radius of 86 mm are installed along the tape path [Fig. S1(*b*)]. While the tape is fed at a speed of several tens to hundreds of millimetres per second, these rollers maintain the tape path and suppress the me­andering motion of the tape in the *x* direction [Fig. 1 ▸(*b*)] to within ±0.1 mm.

**Figure 2:**
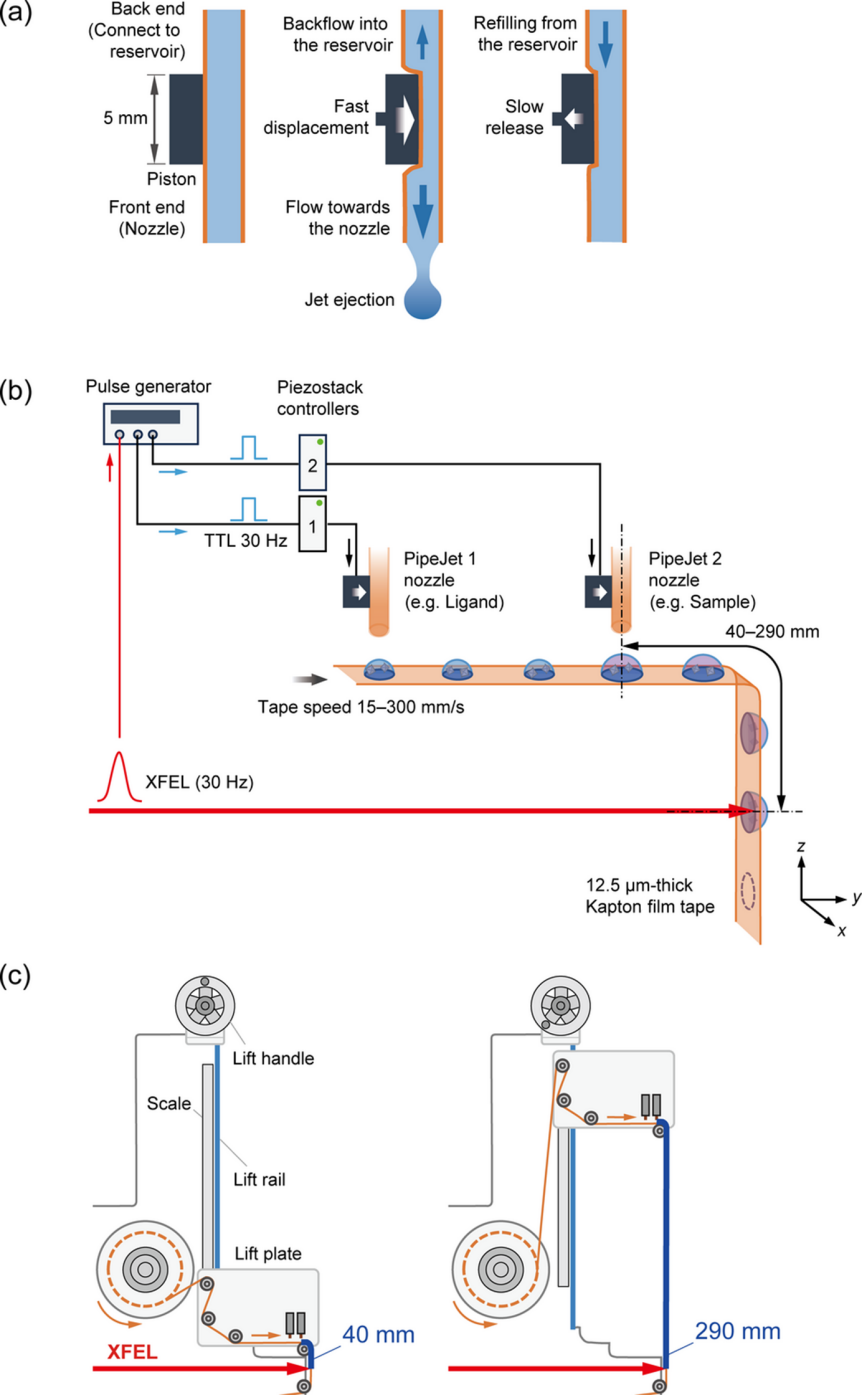
Schematic diagrams of (*a*) the droplet ejection mechanism, (*b*) sample delivery synchronized with XFEL irradiation, and (*c*) control of the distance between the droplet ejection area and XFEL intersecting area.

A commercial piezoelectric injector (PipeJet, Hamilton, https://www.hamiltoncompany.com/pipejet) is used to dis­pense sample droplets onto the tape surface. Fig. 2 ▸(*a*) shows the droplet ejection mechanism of the PipeJet (Streule *et al.*, 2004 ▸). A thin polymer tube with a typical inner diameter (ID) of 200 µm is used as a disposable nozzle that connects the back end to the sample reservoir; tubes with diameters of 125 and 500 µm are also available. A piston connected to a piezostack actuator squeezes the tube over an active area with a length of approximately 5 mm, which displaces the sample solution towards both ends of the tube. The sample solution that is pushed towards the tip is dispensed as nanolitre-scale droplets that form a queue on the tape surface with a constant spacing depending on the tape speed (*e.g.* a spacing of 1 mm corresponds to a tape speed of 30 mm s^−1^). The volume of the sample droplets depends on the displacement and velocity of the piston, the inner diameter of the nozzle, and the viscosity of the samples. For pure water, 125, 200 and 500 µm ID tubes can generate 2–12, 5–18 and 20–75 nl droplets, respectively, at up to 50 Hz maximum frequency (Hamilton, https://www.hamiltoncompany.com/pipejet). Fig. S2(*a*) shows a pure water droplet queue with a volume of 9 nl ejected by the PipeJet with a 200 µm ID tube onto the tape surface of the Kapton film without any water-repellent treatment. Each droplet forms a flattened dome shape with a diameter of about 500 µm and a height of about 90 µm. A commercial 1 ml Terumo syringe is used as the sample reservoir and the nozzle protrudes 2 mm from the rest of the piezoelectric injector [Fig. S2(*b*)]. A simple stirring propeller is mounted onto the sample reservoir to prevent sedimentation of the microcrystals [Figs. S2(*c*) and S2(*d*)]. In the ejection section [Fig. S2(*e*)], the nozzle is aimed at the tape surface, and an antistatic brush helps reduce tape charging.

For mixing experiments, two PipeJet units are mounted on a lift plate: one unit dispenses droplets containing microcrystals, while the other ejects a solution containing a substrate or ligand. The distance between the nozzle tips of PipeJet units 1 and 2 is manually adjusted with respect to the tape speed so that the droplets overlap precisely, usually by moving PipeJet unit 1 along the *y*-axis direction (*e.g.* a distance of 15 mm at a tape speed of 30 mm s^−1^). PipeJet units 1 and 2 are individually mounted onto the lift plate with *x*-, *y*- and *z*-axis micrometer stages and *x*- and *z*-axis micrometer stages, respectively (Fig. S3). Each droplet is dispensed in synchronization with a transistor–transistor logic signal generated by a pulse generator as a trigger signal referenced to the XFEL clock signal at SACLA. The two droplets from the PipeJet units are overlaid and then irradiated with an XFEL pulse after a certain delay time. As shown in Fig. 2 ▸(*c*), the distance from the location where the two droplets are mixed to the XFEL intersecting area can be varied from 40 to 290 mm. The lift plate can be moved up and down along the lift rail over a range of 250 mm by turning the lift handle. The delay time after mixing the two droplets can be adjusted from a minimum of 0.13 s to a maximum of 19.3 s depending on the tape speed and distance.

The XFEL pulses are incident perpendicular to the tape’s broad surface and pass through the tape before reaching the droplets. Positioning the tape surface perpendicular to the XFEL beam facilitates hitting the crystals in the droplets because it negates the effects of droplet height and crystal sedimentation. Additionally, the hit ratio of the XFEL pulses onto the droplets is approximately 100% at a speed of 30 mm s^−1^ because the bottom surface of the droplets is wide, allowing the XFEL pulse and the droplets to intersect easily. However, this approach can introduce background noise depending on the thickness of the tape. Figs. S4 and S5 show a typical diffraction pattern of HEWL crystals using a 12.5 µm-thick Kapton tape. Diffraction rings from the tape appear in a low-angle region near the centre (5–20 Å resolution). However, the intensity of the rings is sufficiently low (∼10^–2^) compared with those of the diffraction spots derived from the HEWL crystals that they would not influence data processing for structural analysis. Solvent scattering from the droplets is also observed, but the influence of background noise is not significant because the height of the droplet generating the scattering is relatively low [Figs. S4(*b*) and S5]. The droplets on the tape surface tend to spread since the tape is not treated to be water repellent [Fig. S2(*a*)]. Assuming the droplets maintain the same contact angle relative to the tape surface, a droplet volume of 10 nl leads to a height of approximately 90 µm, while a volume of 14 nl results in a height of approximately 100 µm, giving a difference of only approximately 10%.

## 3. Time-resolved mixing SFX experiment

### 3.1. Sample preparation

Mixing experiments were conducted using HEWL as the protein crystals and GlcNAc as an inhibitor. HEWL is a glycoside hydro­lase that is widely used in X-ray crystallography as a commercially available model protein and was crystallized by a batch method according to a modified version of the previously described protocol (Nango *et al.*, 2015 ▸; Sugahara *et al.*, 2015 ▸). First, 10 ml of 20 mg ml^−1^ HEWL (FUJIFILM Wako Pure Chemical) dissolved in 0.1 *M* sodium acetate (pH 3.0) was added to an equivalent volume of crystallization buffer [8% (*w*/*v*) polyethyl­ene glycol (PEG) 6000, 4.8 *M* NaCl and 0.1 *M* sodium acetate (pH 3.0)]. The mixture was then equilibrated using a ThermoMixer C (Eppendorf) for 10 min at a rotational speed of 500 rev min^−1^ and a temperature of 12 or 17°C, which resulted in HEWL microcrystals with dimensions of 1 or 3–5 µm, respectively. The 1 and 3–5 µm crystals had crystal densities of approximately 1.1 × 10^10^ and 4.8 × 10^8^ crystals ml^−1^, respectively. The micro­crystals were then harvested by centrifugation at 3000*g* for 5 min at 4°C. The supernatant was replaced with a harvest buffer [1 *M* sodium acetate (pH 3.0), 1.7 *M* NaCl] and the microcrystals were stored at 4°C until use. The 1 µm crystals were diluted at a 1:10 ratio to a final density of ∼1.1 × 10^9^ crystals ml^−1^ using the harvest buffer before SFX measurements, while the 3–5 µm crystals were used as is. These densities correspond to 11000 crystals per drop (10 nl) for the 1 µm crystal and 4800 crystals per drop for the 3–5 µm crystal. Using a 1.5 µm XFEL beam, this is expected to result in diffraction patterns from a single crystal per image. The protein concentration for each crystal suspension was estimated from the number of protein mol­ecules in the unit cell and the volume of the crystal.

For the inhibitor, 50 mg ml^−1^ (226 m*M*) and 100 mg ml^−1^ (452 m*M*) of GlcNAc were dissolved in water or various buffers including PEG. HEWL crystal droplets were then merged with the different inhibitor droplets at different concentrations and with different buffer types, and a microscope was used to check visually whether the crystal and inhibitor droplets had mixed properly. The inhibitor dissolved in water did not seem to mix well with the crystal droplets. Thus, the inhibitor dissolved in a buffer of 1 *M* NaCl and 15% PEG 4000 was selected for the mixing experiments. The concentration of the 226 m*M* GlcNAc solution was approximately 100 and 1000 times the molar excess relative to the undiluted microcrystal suspension and tenfold-diluted suspension, respectively. The inhibitor solution was filtered through a 0.22 µm filter before measurements.

### 3.2. Diffraction data collection

Data were collected in Experimental Hutch 3 of SACLA Beamline 2 (Ishikawa *et al.*, 2012 ▸; Tono *et al.*, 2019 ▸) using XFEL pulses with a photon energy of 10 keV, a pulse duration of 10 fs or less, and a pulse energy of 370 µJ on average before the first mirror in Optics Hutch 1; each XFEL pulse contained approximately 2 × 10^11^ photons per pulse at a repetition rate of 30 Hz. The X-ray beam was focused to an FWHM diameter of 1.5 µm by two X-ray ellipsoidal mirrors in the Kirkpatrick–Baez geometry. To avoid the parasitic scattering of X-rays by air, a collimator was mounted on the CoT and helium gas was introduced at a flow rate of 1.4 ml min^−1^. Measurements were performed at a temperature of 25–26°C and a relative humidity of 30–40%. Diffraction patterns were recorded using an MPCCD detector (Phase III) (Kameshima *et al.*, 2014 ▸) with eight sensor modules at a sample-to-detector distance of 70 mm. Implementation details for operating the CoT, such as control software, are shown in Fig. S6.

Droplets were dispensed at a piezostack stroke of 5% and a stroke velocity of 120 µm ms^−1^. A nozzle with an inner diameter of 200 µm was mounted onto each PipeJet unit. One PipeJet unit ejected an inhibitor droplet containing GlcNAc, which was overlaid by an equal volume droplet containing HEWL crystals ejected by the second PipeJet unit. Individually, the crystal and inhibitor droplets had an average volume of 10–14 nl with a diameter of about 520–580 µm and a height of about 90–100 µm on the tape surface. The merged droplet reached a diameter of about 660–740 µm and a height of about 115–130 µm. The tape speed was set to 30 mm s^−1^, while the distance between the point at which the second droplet is ejected and the XFEL irradiation point was varied in the range of 40–290 mm. Thus, the mixing time between the merging of the two droplets and XFEL irradiation was varied in the range of 1.3–9.7 s. Finally, 13 datasets were obtained under different conditions, including the resting state of the HEWL crystals without the addition of inhibitor droplets. These data were collected during 36 h of beamtime under proposal No. 2023A8008.

### 3.3. Data processing and structure determination

Diffraction images were filtered to extract ‘hit images’ that included diffraction patterns from the crystals using *Cheetah* (Barty *et al.*, 2014 ▸), which was modified for processing SFX data at SACLA (Nakane *et al.*, 2016 ▸). A hit image was defined as containing a minimum of 20 Bragg peaks. The detector geometry was refined by *geoptimizer* in *CrystFEL* (Version 0.10.2) (White *et al.*, 2013 ▸). Autoindexing and integration were performed using *CrystFEL*. The resting structure of lysozyme was solved using the molecular replacement method in the software *Phaser* (McCoy *et al.*, 2007 ▸). The PDB model 6jzi (Shimazu *et al.*, 2019 ▸) from the Protein Data Bank was applied as a reference after the water molecules and metal ions were removed. Further refinement of the structure was conducted using *Phenix* (Adams *et al.*, 2010 ▸) and *COOT* (Emsley *et al.*, 2010 ▸). Finally, *MOLPROBITY* (Chen *et al.*, 2010 ▸) was used for data validation. The differences in the electron-density maps between the mixing data and resting state data were calculated with the *CCP4 toolkit* (Agirre *et al.*, 2023 ▸). All structure images were created using *PyMol* (Schrödinger & DeLano, 2020 ▸).

For the indexing algorithm, *XGANDALF* (extended gradient descent algorithm for lattice finding) implemented in *CrystFEL* was chosen (Gevorkov *et al.*, 2019 ▸), which is specifically designed for still diffraction images, such as those collected in serial femtosecond crystallography. It converts detected Bragg peaks into reciprocal-space vectors and applies a gradient-descent optimization to identify lattice basis vectors that best explain the observed reflections. Unlike traditional algorithms [*e.g.MOSFLM* (Duisenberg, 1992 ▸) or *DirAx* (Powell, 1999 ▸)], *XGANDALF* does not require prior unit-cell information and performs well even with a limited number of spots. It can also detect multiple lattices per image, making it suitable for cases where more than one crystal is hit by an XFEL pulse.

## 4. Results

To demonstrate the performance of the CoT, we conducted time-resolved SFX experiments mixing HEWL crystals of different sizes and the inhibitor GlcNAc. Two concentrations of the inhibitor were prepared and first dispensed as droplets of 10–14 nl on the tape. Subsequently, HEWL crystals were ejected into the first droplet in the same volume, which was basically mixed by diffusion. The equilibration/reaction time was adjusted by moving the lift plate on the CoT.

The 1 µm HEWL crystals were mixed with 226 m*M* GlcNAc and measured at delay times of 1.3, 5.0, 7.5 and 9.7 s, or mixed with 452 m*M* GlcNAc and measured at delay times of 1.3, 2.5, 4.0 and 5.0 s. The hit ratio was 14–31% and the indexed rate was 77% on average.

The 3–5 µm HEWL crystals were mixed with 226 m*M* GlcNAc and measured at delay times of 2.0, 5.0 and 9.7 s. The hit ratio was 40–54% and the indexed rate was 80% on average.

Each dataset comprised 10000–24000 indexed images. Refined structural models were determined with a resolution of 1.68–1.82 Å. One dataset typically required approximately one hour to collect and used one roll of tape spooling out at 30 mm s^−1^. Data collection proceeded smoothly and was often only interrupted if/when tape replacement was required. This setup is basically intended to be operated by the user. Thus, the user needs to replace a pair of reel-to-reel tapes. Tape replacement typically takes about 5 min, although it may take longer initially.

In the experiments, 10–14 nl of the crystal suspension was used per droplet. Thus, the sample consumption for the 1 µm HEWL crystals was 0.1 µg per diffraction image, or 0.5–1.0 mg for 10000 indexed images. The sample consumption for the 3–5 µm HEWL crystals was 2 µg per diffraction image, or 12–17 mg for 10000 indexed images.

**Figure 3:**
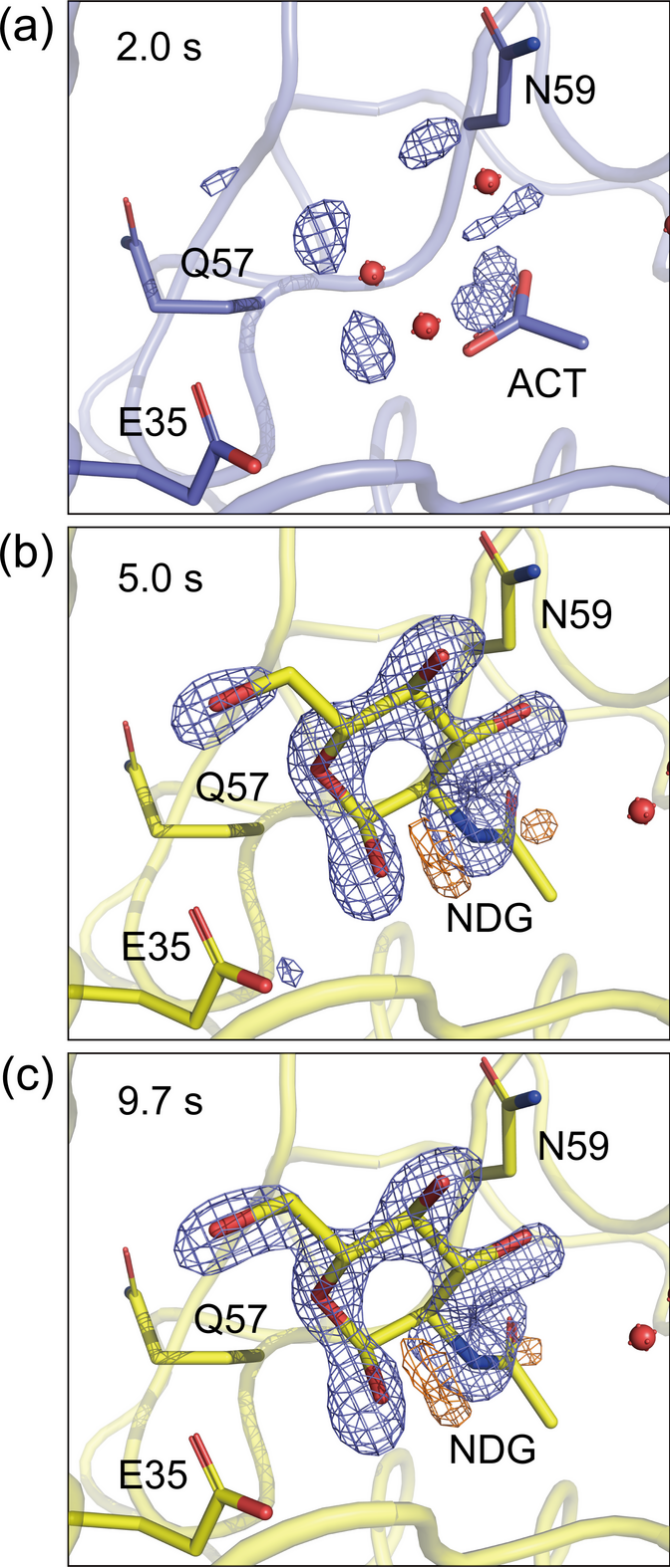
*F*_o (bound)_ − *F*_o (resting)_ difference electron-density maps obtained by mixing 3–5 µm HEWL crystals with 226 m*M* GlcNAc at mixing times of (*a*) 2.0 s, (*b*) 5.0 s and (*c*) 9.7 s. The maps are contoured at 5.00σ. The HEWL structure in the absence of GlcNAc is shown with blue carbon atoms, while the GlcNAc-bound HEWL structure is shown with yellow carbon atoms. The positive and negative electron-density peaks are depicted in slate blue and orange, respectively.

**Figure 4:**
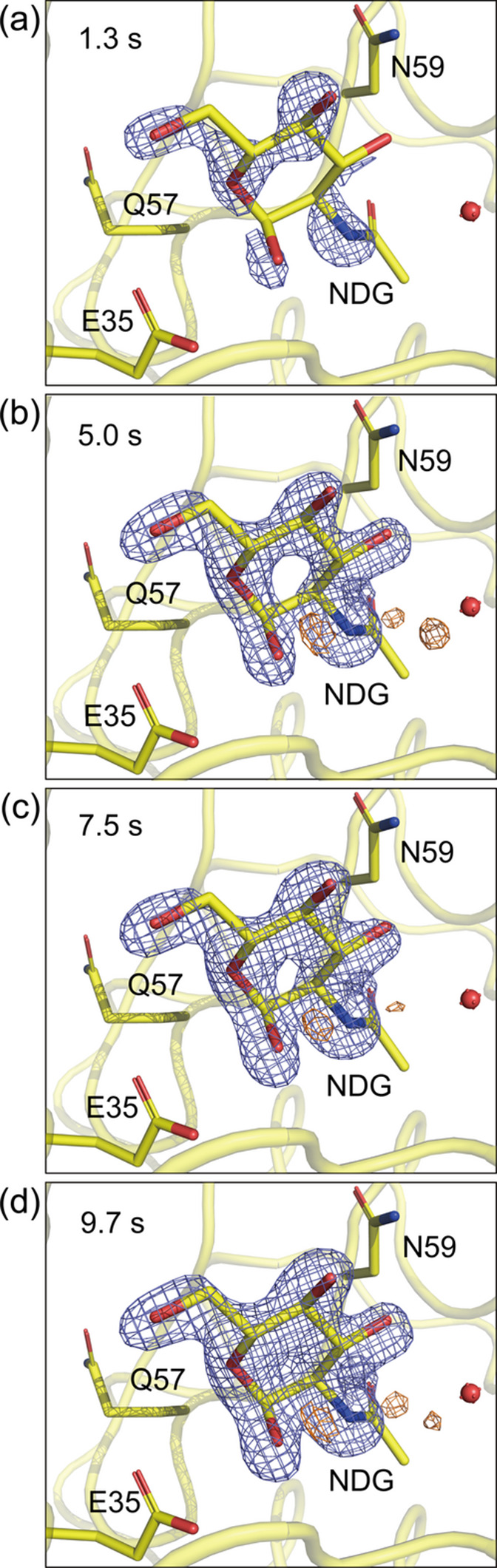
*F*_o (bound)_ − *F*_o (resting)_ difference electron-density maps obtained by mixing 1 µm HEWL crystals with 226 m*M* GlcNAc at mixing times of (*a*) 1.3 s, (*b*) 5.0 s, (*c*) 7.5 s and (*d*) 9.7 s. The maps are contoured at 6.00σ. The GlcNAc-bound HEWL structure is shown with yellow carbon atoms. The positive and negative electron-density peaks are depicted in slate blue and orange, respectively.

**Figure 5:**
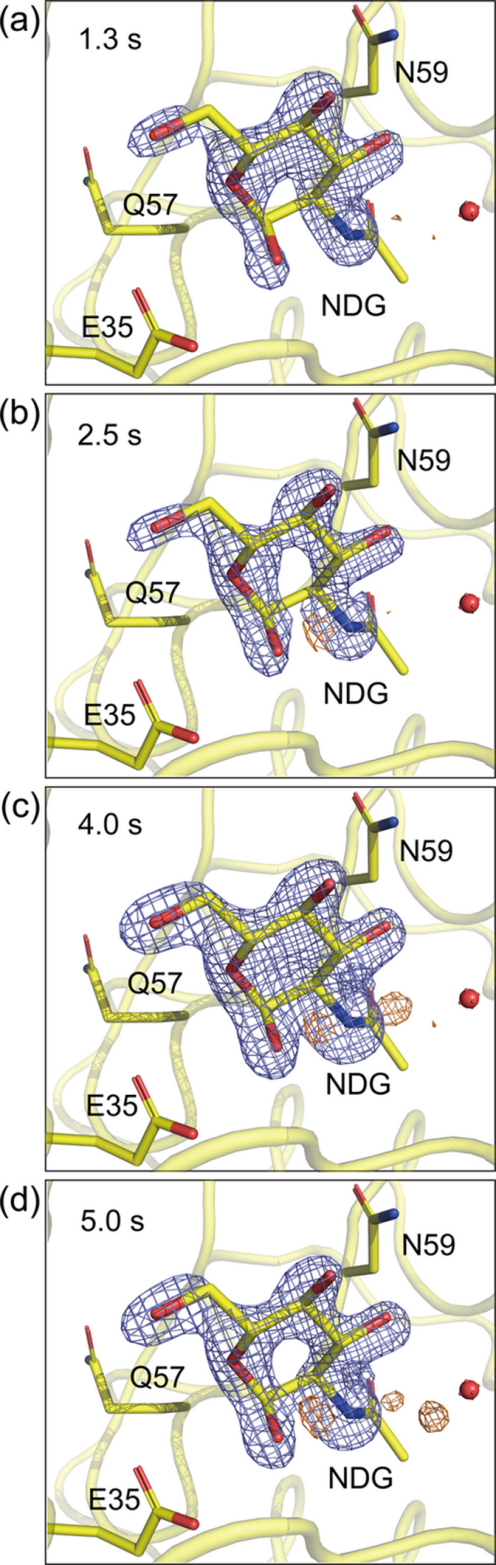
*F*_o (bound)_ − *F*_o (resting)_ difference electron-density maps obtained by mixing 1 µm HEWL crystals with 452 m*M* GlcNAc at mixing times of (*a*) 1.3 s, (*b*) 2.5 s, (*c*) 4.0 s and (*d*) 5.0 s. The maps are contoured at 6.00σ. The GlcNAc-bound HEWL structure is shown with yellow carbon atoms. The positive and negative electron-density peaks are depicted in slate blue and orange, respectively.

The resting state structures of the 1 and 3–5 µm HEWL crystals both show an acetate ion and several water molecules in the active site (Table 1 ▸). To visualize the binding process of the inhibitor, difference electron-density (DED) maps were calculated by subtracting the resting state data from the time-resolved mixing SFX data. The *F*_o_ − *F*_c_ maps show not only the electron densities corresponding to the inhibitor but also the original acetate ion and water molecules, which prevents an accurate estimation of the inhibitor binding ratio. Therefore, the evaluation of the inhibitor binding ratio was based on the intensity of the peaks in the DED maps. Fig. 3 ▸ shows the DED maps of 3–5 µm HEWL crystals mixed with 226 m*M* GlcNAc. At a mixing time of 2.0 s, the inhibitor was partially bound to the active site. The inhibitor binding ratio increased at a mixing time of 5.0 s but then remained stable as the mixing time increased further to 9.7 s, as presented in Table 2 ▸. Fig. 4 ▸ shows the DED maps of 1 µm HEWL crystals mixed with 226 m*M* GlcNAc, which indicates that the inhibitor was partially bound to the active site at the mixing time of 1.3 s. Despite the shorter mixing time compared with the 3–5 µm HEWL crystals, the inhibitor binding ratio was the same, as given in Table 3 ▸. In addition, the inhibitor binding ratio gradually increased over time. Fig. 5 ▸ shows the DED maps of 1 µm HEWL crystals mixed with 452 m*M* GlcNAc. At a mixing time of 1.3 s, the peak intensity at the active site is higher than that shown in Fig. 4 ▸, which suggests that increasing the inhibitor concentration has increased the inhibitor binding ratio. As given in Table 4 ▸, the inhibitor binding ratio also gradually increased with the mixing time. In summary, we successfully observed an increase in the inhibitor binding ratio according to the crystal size and mixing time.

## 5. Discussion

The main differences between the CoT and the DOT method of Fuller *et al.* (2017 ▸) are that in the CoT the XFEL pulses pass through the tape and piezoelectric injectors are used rather than acoustic droplets (‘injectors’) to dispense droplets. In the experiments performed at SACLA in 2023, the volume of the droplets was three to ten times larger than those typically obtained with the DOT method, but the sample volume can be reduced. After conducting tests, we found that the volume depends on the condition of the device and nozzle. Subsequently, we successfully ejected droplets of 4 to 6 nl using a 200 µm nozzle with a solution containing crystals. With a 125 µm nozzle, we were able to dispense droplets including buffer or crystals of 1–2 nl.

One concern is that the tape may be punctured by the intense XFEL pulses, which matters mostly for systems that clean and reuse the same tape and less so for reel-to-reel systems. In fact, we observed small holes in the tape surface after XFEL irradiation that are fully consistent with the X-ray beam size and alignment setup (Fig. S7). However, the tape was driven stably without any problems during data collection, indicating that small perforations drilled through by the 1.5 µm XFEL beam diameter did not compromise the CoT. Although the tape was replaced after a single use, it can potentially be reused by shifting the XFEL intersecting area and installing a cleaning bath to remove salts and crystals. The background noise induced by the tape was relatively low because the tape was only 12.5 µm thick (Fig. S4). This is much thinner than the sample streams from the high-viscosity sample injector typically used at SACLA, which are 75–100 µm in diameter. The thickness of the tape was set to 12.5 µm to achieve low background noise while balancing manufacturing costs and handling.

In the experiments, the crystal and inhibitor droplets had the same volumes, so mixing them halved the inhibitor concentrations from 226 to 113 m*M* and from 452 to 226 m*M*. Given that GlcNAc has an affinity constant of 47.6 m*M* (Kumagai *et al.*, 1992 ▸), these concentrations are sufficient. Inhibitor binding was observed after a mixing time of 1.3 s under all conditions, and the bound inhibitors showed different occupancies depending on the concentration and crystal size.

According to our knowledge, this is the first report to perform time-resolved mixing SFX experiments using different HEWL crystal sizes and inhibitor concentrations. However, to give a full characterization of the time-resolved crystallography parameter space for enzyme reactions, a comprehensive study should include (i) a range of homogenous crystal size slurries (*e.g.* 0.5, 1, 2, 3, 5, 7.5 and 10 µm dimensions), (ii) a range of ligand concentrations (*e.g.* from 0.1× to 10×) related to the known *K*_M_, *K*_I_ or *K*_D_ values measured in solutions and correlated to crystals, (iii) known or experimentally determined ligand diffusion times in water and mother liquor, as well as through crystal structures, (iv) a range of temperatures, and (v) more than one space group and crystal packing condition across the same reaction coordinate.

The diffusion time of small molecules into crystals is known to depend on the crystal size and ligand concentration, which we successfully demonstrated experimentally using the CoT. The CoT is capable of observing mixing times of 0.1–19.3 s, and it is possible that binding could have taken place at an earlier mixing time. To achieve the earliest mixing time, the tape needs to be driven at a speed of 300 mm s^−1^. We tested dispensing droplets onto the tape while moving it at 30–300 mm s^−1^, and the droplets were transported stably (Movies S1–S4 in the supporting information). A long delay time raises concerns about droplets drying up. We ejected pure water droplets of 5 nl onto the tape and observed them. The droplets required one minute to evaporate completely (Movie S5). Therefore, mixing times of 0.1–19.3 s appear to be feasible. However, we did not perform TR-SFX experiments under such conditions because of the limited beamtime available at SACLA. Butryn *et al.* (2021 ▸) collected time-resolved SFX data at a mixing and equilibration time of 0.6 s using 3–5 µm HEWL crystals with 16.7 m*M* GlcNAc as the final concentration. They injected multiple picolitre-volume droplets of the inhibitor solution into a ∼3 nl crystal slurry droplet to create turbulence, which resulted in a higher mixing efficiency. In this study, we induced rapid diffusion by increasing the inhibitor concentration by 3–14 times compared with that used by Butryn *et al.* (43.7 m*M*) to achieve inhibitor binding at a similar mixing time. If increasing the ligand concentration is difficult because of constraints such as its solubility, alternative approaches to accelerate ligand diffusion into crystals would be necessary, such as turbulent mixing or heating. The volume of droplets also affects the diffusion time. Therefore, reducing the volume is effective for rapid diffusion, and the use of a small nozzle such as 125 µm is recommended.

In TR-SFX, sample consumption is a critical concern because protein and ligand samples are often scarce and/or expensive. Moreover, for a TR-SFX reaction coordinate, data need to be collected at multiple time points to visualize the structural changes over time. We summarize each sample consumption used in the mixing experiments in Tables S1–S3. With the CoT, collecting 10000 indexed images of 3–5 µm HEWL crystals consumed 12–21 mg of HEWL. Meanwhile, Butryn *et al.* (2021 ▸) reported that they consumed 6.5–27 mg of HEWL to collect 10000 indexed images of the same crystal size. When we used 1 µm HEWL crystals, we only used 0.4–1 mg of HEWL to obtain 10000 indexed images, which indicates that the sample consumption can be decreased by reducing the crystal size. Furthermore, we used a droplet volume of 10–14 nl in the experiments, which can also be reduced to improve efficiency. The droplet volume ejected by the PipeJet unit depends on the size of the nozzle and the composition and viscosity of the solution. Offline tests with the CoT using a nozzle size of 200 µm showed that it could dispense GlcNAc droplets with a volume of 3–6 nl and HEWL crystal droplets with a volume of 4–6 nl by optimizing the parameters for droplet ejection. A thinner nozzle of 125 µm can be used to reduce the droplet volume further to 1–2 nl.

## 6. Conclusion

In this study, we have developed a compact tape-driven sample delivery system, CoT. Piezoelectric injectors were used to dispense sample droplets, and XFEL pulses were perpendicularly irradiated onto the tape to facilitate alignment of the intersection of the sample droplets with the XFEL. The tape transport speed and the distance between the droplet ejection area and the XFEL intersecting area were designed to be variable, allowing a mixing time range from 0.1 to 19.3 s.

We have successfully demonstrated that the CoT can be employed for time-resolved mixing SFX experiments using HEWL crystals of different crystal sizes and solution concentrations. Under all conditions, the inhibitor was observed to bind within a mixing time of 1.3 s. Reducing the HEWL crystal size to 1 µm decreased sample consumption to less than 1 mg for 10000 indexed images. The method is also applicable to light-triggered pump–probe SFX because a pump laser can be introduced into the setup.

The CoT is expected to reduce the sample consumption of time-resolved SFX experiments and to contribute greatly to the dynamic structural analysis of various proteins in the future.

## 7. Related literature

For further literature related to the supporting information, see Joti *et al.* (2015 ▸).

## Supplementary Material

Additional figures and tables. DOI: 10.1107/S1600576726000063/te5159sup1.pdf

CIF files for PDB IDs: 9v3d, 9v3g, 9v3h, 9v3i, 9v3j, 9v3k, 9v3l, 9v3m, 9v3n, 9v3o, 9v3p, 9v3q and 9v3r. DOI: 10.1107/S1600576726000063/te5159sup2.zip

Movie S1, 9nl, 30mm per sec. DOI: 10.1107/S1600576726000063/te5159sup3.avi

Movie S2, 9nl, 60 mm per sec. DOI: 10.1107/S1600576726000063/te5159sup4.avi

Movie S3, 9nl, 150 mm per sec. DOI: 10.1107/S1600576726000063/te5159sup5.avi

Movie S4, 9nl, 300 mm per sec. DOI: 10.1107/S1600576726000063/te5159sup6.avi

Movie S5, 5nl. DOI: 10.1107/S1600576726000063/te5159sup7.avi

Raw diffraction data deposited in the CXIDB entry 236: https://doi.org/10.11577/3014306

PDB reference: 9v3d

PDB reference: 9v3g

PDB reference: 9v3h

PDB reference: 9v3i

PDB reference: 9v3j

PDB reference: 9v3k

PDB reference: 9v3l

PDB reference: 9v3m

PDB reference: 9v3n

PDB reference: 9v3o

PDB reference: 9v3p

PDB reference: 9v3q

PDB reference: 9v3r

## Figures and Tables

**Table 1:** Crystallographic statistics of 1 and 3–5 µm HEWL crystals without inhibitor

	Crystal size	
	1 µm	3–5 µm
Tape speed (mm s^−1^)	30	30
PDB ID	9v3g	9v3d
Hit rate (%)	30.70	53.60
Crystals per frame†	0.367	0.767
Indexing rate (%)	81.02	75.24
Effective indexing rate (%)‡	24.87	40.33
Indexed images	23501	12213
Unit cell (Å, °)	79.50, 79.50, 38.20, 90.00, 90.00, 90.00	79.45, 79.45, 38.15, 90.00, 90.00, 90.00
Resolution (Å)§	39.68–1.74 (1.76–1.74)	39.68–1.68 (1.70–1.68)
Total reflections§	3804235 (25922)	2931509 (16513)
Unique reflections§	13099 (622)	14477 (697)
Redundancy§	290.4 (41.70)	202.5 (23.70)
Completeness (%)§	100.0 (100.00)	100.0 (100.00)
*I*/σ(*I*)§	8.49 (1.18)	7.46 (1.16)
Wilson *B* factor	29.64	30.47
*R*_split_ (%)§	8.9 (96.43)	13.2 (63.21)
*CC*_1/2_ §	0.989 (0.4771)	0.970 (0.6994)

Refinement		
Reflections used in refinement	13058	14446
Reflections used for *R*_free_	653	723
*R*_work_	0.1736	0.1895
*R*_free_	0.1966	0.2247
Occupancy of inhibitor (%)	–	–
No. of non-hydrogen atoms		
Protein	1021	1021
Ligands	7	7
Solvent	77	77
RMS deviations		
Bond lengths (Å)	0.003	0.007
Bond angles (°)	0.67	0.96
Ramachandran		
Favoured (%)	98.43	98.43
Allowed (%)	1.57	1.57
Outliers (%)	0	0
Rotamer outliers (%)	0.93	0.93
Clashscore	0.99	0.99
*B* factors		
Average (Å^2^)	27.75	23.39
Protein (Å^2^)	26.81	22.54
Ligands/ion (Å^2^)	35.24	27.97
Solvent	39.48	34.18

† Estimated using the ‘Hit rate calculator’ available at https://www.desy.de/~twhite/crystfel/hitrate.html.

‡ Effective indexing rate refers to the ratio of successfully indexed diffraction patterns to the total number of acquired frames.

§ Values in parentheses are for the highest-resolution shell.

**Table 2:** Crystallographic statistics of 3–5 µm HEWL crystals bound with 226 m*M* GlcNAc

	Mixing time		
	2 s	5 s	9.7 s
Tape speed (mm s^−1^)	30	30	30
Droplet transport distance† (mm)	60	150	290
PDB ID	9v3h	9v3i	9v3j
Hit rate (%)	50.40	45.00	39.80
Crystals per frame‡	0.702	0.597	0.507
Indexing rate (%)	79.50	82.32	81.87
Effective indexing rate (%)§	40.07	37.04	32.58
Indexed images	10062	20383	16067
Unit cell (Å, °)	79.45, 79.45, 38.15, 90.00, 90.00, 90.00	79.45, 79.45, 38.15, 90.00, 90.00, 90.00	79.45, 79.45, 38.15, 90.00, 90.00, 90.00
Resolution (Å)¶	39.68–1.68 (1.70–1.68)	39.68–1.68 (1.70–1.68)	39.68–1.68 (1.70–1.68)
Total reflections¶	2356211 (13179)	4859263 (26654)	2715055 (14754)
Unique reflections¶	14489 (702)	14489 (702)	14488 (702)
Redundancy¶	162.6 (18.80)	335.4 (38.00)	187.4 (21.00)
Completeness (%)¶	100.0 (100.00)	100.0 (100.00)	100.0 (100.00)
*I*/σ(*I*)¶	6.48 (1.10)	9.52 (1.65)	7.31 (1.19)
Wilson *B* factor	30.82	30.02	30.03
*R*_split_ (%)¶	14.9 (80.12)	9.9 (51.85)	13.6 (69.40)
*CC*_1/2_ ¶	0.962 (0.5975)	0.984 (0.7500)	0.969 (0.7053)

Refinement			
Reflections used in refinement	14446	14440	14439
Reflections used for *R*_free_	722	723	722
*R*_work_	0.1947	0.1853	0.1869
*R*_free_	0.224	0.2212	0.223
Occupancy of inhibitor (%)	73	74	73
No. of non-hydrogen atoms			
Protein	1025	1025	1025
Ligands	18	18	18
Solvent	64	67	67
RMS deviations			
Bond lengths (Å)	0.006	0.004	0.013
Bond angles (°)	0.98	0.73	1.41
Ramachandran			
Favoured (%)	97.64	98.43	97.64
Allowed (%)	2.36	1.57	2.36
Outliers (%)	0	0	0
Rotamer outliers (%)	0.93	0.93	0.93
Clashscore	1.47	0.49	0.98
*B* factors			
Average (Å^2^)	24.90	24.16	23.62
Protein (Å^2^)	24.17	23.48	22.98
Ligands/ion (Å^2^)	30.09	24.93	24.8
Solvent	35.01	34.26	33.11

† The distance from the location where the two droplets are mixed at the XFEL intersecting area.

‡ Estimated using the ‘Hit rate calculator’ available at https://www.desy.de/~twhite/crystfel/hitrate.html.

§ Effective indexing rate refers to the ratio of successfully indexed diffraction patterns to the total number of acquired frames.

¶ Values in parentheses are for the highest-resolution shell.

**Table 3:** Crystallographic statistics of 1 µm HEWL crystals bound with 226 m*M* GlcNAc

	Mixing time			
	1.3 s	5 s	7.5 s	9.7 s
Tape speed (mm s^−1^)	30	30	30	30
Droplet transport distance†	40 mm	150 mm	225 mm	290 mm
PDB ID	9v3k	9v3l	9v3m	9v3n
Hit rate (%)	25.77	23.50	24.20	14.40
Crystals per frame‡	0.298	0.268	0.277	0.156
Indexing rate (%)	80.84	64.07	81.98	80.19
Effective indexing rate (%)[Table-fn tfn10]	20.83	15.06	19.84	11.55
Indexed images	39020	16967	11967	17407
Unit cell (Å, °)	79.50, 79.50, 38.20, 90.00, 90.00, 90.00	79.50, 79.50, 38.20, 90.00, 90.00, 90.00	79.50, 79.50, 38.20, 90.00, 90.00, 90.00	79.50, 79.50, 38.20, 90.00, 90.00, 90.00
Resolution (Å)[Table-fn tfn11]	39.68–1.72 (1.74–1.72)	39.68–1.75 (1.78–1.75)	39.68–1.77 (1.80–1.77)	39.68–1.78 (1.81–1.78)
Total reflections[Table-fn tfn11]	6390960 (33815)	2927612 (19507)	2152845 (19216)	2882272 (18231)
Unique reflections[Table-fn tfn11]	13560 (661)	12892 (611)	12477 (620)	12281 (613)
Redundancy[Table-fn tfn11]	471.3 (51.20)	227.1 (31.90)	172.5 (31.00)	234.7 (29.70)
Completeness (%)[Table-fn tfn11]	100.0 (100.00)	100.0 (100.00)	100.0 (100.00)	100.0 (100.00)
*I*/σ(*I*)[Table-fn tfn11]	10.63 (1.03)	7.56 (1.02)	6.85 (1.04)	8.43 (1.32)
Wilson *B* factor	29.81	28.58	29.23	28.71
*R*_split_ (%)[Table-fn tfn11]	6.9 (101.02)	10.8 (105.96)	11.5 (99.77)	9.1 (85.43)
*CC*_1/2_ [Table-fn tfn11]	0.994 (0.4427)	0.985 (0.4146)	0.980 (0.4291)	0.989 (0.4833)

Refinement				
Reflections used in refinement	13518	12850	12437	12239
Reflections used for *R*_free_	677	643	622	612
*R*_work_	0.1774	0.1788	0.1821	0.1672
*R*_free_	0.2056	0.1988	0.2185	0.1882
Occupancy of inhibitor (%)	68	74	77	79
No. of non-hydrogen atoms				
Protein	1025	1025	1025	1015
Ligands	18	18	18	18
Solvent	64	64	64	67
RMS deviations				
Bond lengths (Å)	0.003	0.005	0.003	0.007
Bond angles (°)	0.67	0.84	0.67	0.89
Ramachandran				
Favoured (%)	98.43	99.21	98.43	99.21
Allowed (%)	1.57	0.79	1.57	0.79
Outliers (%)	0	0	0	0
Rotamer outliers (%)	1.87	0.93	0.93	0.95
Clashscore	0.98	1.47	0.49	0.99
*B* factors				
Average (Å^2^)	28.02	27.19	26.61	27.35
Protein (Å^2^)	27.27	26.65	26.10	26.73
Ligands/ion (Å^2^)	37.63	29.67	28.96	28.27
Solvent	37.34	35.14	34.22	36.56

† The distance from the location where the two droplets are mixed at the XFEL intersecting area.

‡ Estimated using the ‘Hit rate calculator’ available at https://www.desy.de/~twhite/crystfel/hitrate.html.

§ Effective indexing rate refers to the ratio of successfully indexed diffraction patterns to the total number of acquired frames.

¶ Values in parentheses are for the highest-resolution shell.

**Table 4:** Crystallographic statistics of 1 µm HEWL crystals bound with 452 m*M* GlcNAc

	Mixing time			
	1.3 s	2.5 s	4 s	5 s
Tape speed (mm s^−1^)	30	30	30	30
Droplet transport distance† (mm)	40	75	120	150
PDB ID	9v3o	9v3p	9v3q	9v3r
Hit rate (%)	25.70	24.88	21.50	15.20
Crystal per frame‡	0.297	0.286	0.242	0.165
Indexing rate (%)	82.36	82.45	73.96	78.74
Effective indexing rate (%)§	21.17	20.51	15.90	11.97
Indexed images	15331	12438	12723	17626
Unit cell	79.50, 79.50, 38.20, 90.00, 90.00, 90.00	79.50, 79.50, 38.20, 90.00, 90.00, 90.00	79.50, 79.50, 38.20, 90.00, 90.00, 90.00	79.50, 79.50, 38.20, 90.00, 90.00, 90.00
Resolution (Å)¶	39.68–1.80 (1.83–1.80)	39.68–1.81 (1.84–1.81)	39.68–1.80 (1.83–1.80)	39.68–1.82 (1.85–1.82)
Total reflections¶	2451958 (20282)	1782793 (16506)	1901023 (14302)	1689033 (15393)
Unique reflections¶	11866 (570)	11685 (572)	11866 (570)	11492 (552)
Redundancy¶	206.6 (35.60)	152.6 (28.90)	160.2 (25.10)	147.0 (27.90)
Completeness (%)¶	100.0 (100.00)	100.0 (100.00)	100.0 (100.00)	100.0 (100.00)
*I*/σ(*I*)¶	7.46 (1.15)	6.66 (1.18)	6.80 (1.13)	6.62 (1.12)
Wilson *B* factor	29.49	28.82	29.02	28.59
*R*_split_ (%)¶	10.2 (91.35)	12.1 (94.09)	11.4 (98.65)	12.1 (96.06)
*CC*_1/2_ ¶	0.985 (0.4231)	0.979 (0.4079)	0.980 (0.4280)	0.979 (0.4353)

Refinement				
Reflections used in refinement	11825	11645	11823	11451
Reflections used for *R*_free_	592	583	592	573
*R*_work_	0.1712	0.1728	0.1773	0.1727
*R*_free_	0.2082	0.2218	0.2161	0.1947
Occupancy of inhibitor (%)	75	77	81	79
No. of non-hydrogen atoms				
Protein	1025	1025	1025	1025
Ligands	18	18	18	18
Solvent	64	64	64	64
RMS deviations				
Bond lengths (Å)	0.003	0.006	0.003	0.006
Bond angles (°)	0.71	0.92	0.66	0.86
Ramachandran				
Favoured (%)	99.21	99.21	99.21	98.43
Allowed (%)	0.79	0.79	0.79	1.57
Outliers (%)	0	0	0	0
Rotamer outliers (%)	0.93	0.93	0.93	0.93
Clashscore	1.47	1.47	0.98	0.98
*B* factors				
Average (Å^2^)	28.83	28.00	26.86	27.14
Protein (Å^2^)	28.17	27.36	26.39	26.72
Ligands/ion (Å^2^)	35.72	33.26	26.95	26.6
Solvent	37.49	36.76	34.21	33.97

† The distance from the location where the two droplets are mixed at the XFEL intersecting area.

‡ Estimated using the ‘Hit rate calculator’ available at https://www.desy.de/~twhite/crystfel/hitrate.html.

§ Effective indexing rate refers to the ratio of successfully indexed diffraction patterns to the total number of acquired frames.

¶ Values in parentheses are for the highest-resolution shell.

## Data Availability

Coordinates and structure factors that were generated during the course of this study have been deposited in the Protein Data Bank with accession codes 9v3d, 9v3g, 9v3h, 9v3i, 9v3j, 9v3k, 9v3l, 9v3m, 9v3n, 9v3o, 9v3p, 9v3q and 9v3r. Raw diffraction data have been deposited in the CXIDB, entry No. 236 (https://doi.org/10.11577/3014306).
