# Supplementary material for: Compact tape-driven sample delivery system for serial femtosecond crystallography
Source: J Appl Crystallogr. 2026 Feb 9;59(Pt 2):291–302. doi: 10.1107/S1600576726000063 (PMC13060467; doi:10.1107/S1600576726000063)
Supplement: Supplementary file 1 [file j-59-00291-sup1.pdf]

JOURNAL OF APPLIED CRYSTALLOGRAPHY

Volume 59 (2026)

Supporting information for article:

Compact tape-driven sample delivery system for serial femtosecond crystallography

Jungmin Kang, Yoshiaki Shimazu, Fangjia Luo, Ayumi Yamashita, Tomoyuki Tanaka, Yuichi Inubushi, Kensuke Tono, Nipawan Nuemket, Allen M. Orville, So Iwata, Eriko Nango and Makina Yabashi

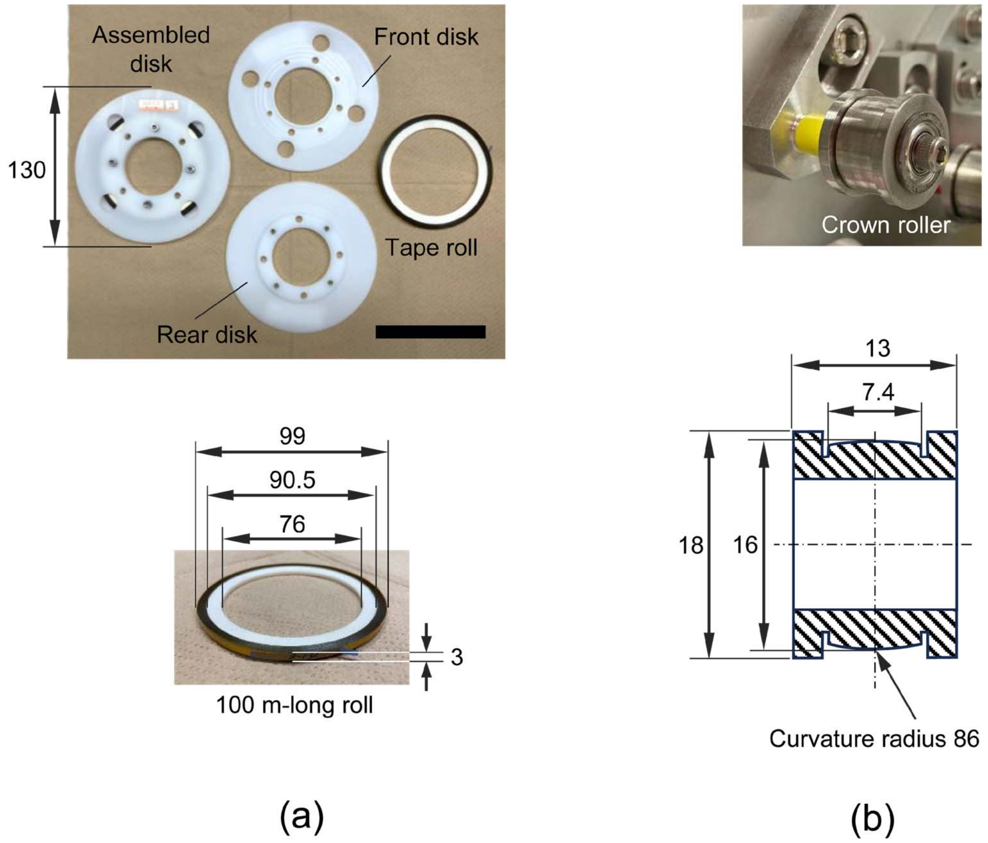

Figure S1 Details of the CoT system components: (a) Photographs of the tape cartridge disc with a typical tape roll. The scale bar in the right-hand corner represents 100 mm. (b) Design of the crown roller. Units are in millimetres.

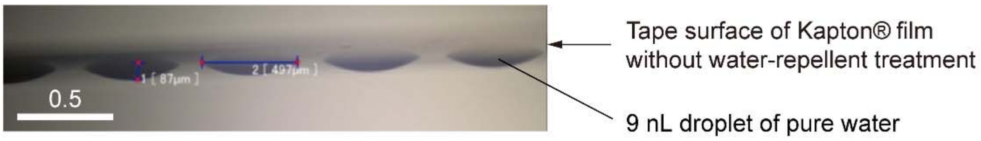
Tape surface of Kapton® film
without water-repellent treatment
9 nL droplet of pure water
0.5
1 [87μm]
2 [497μm]

(a)

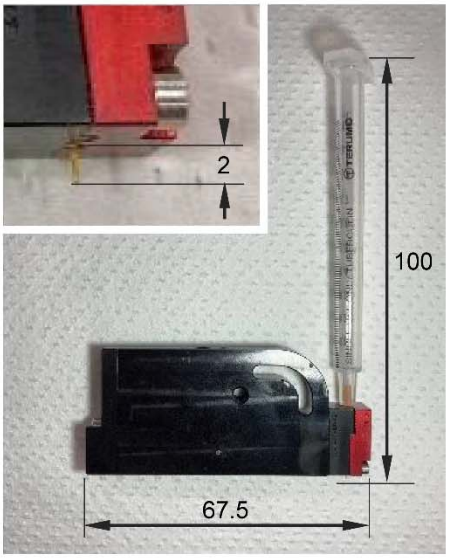
2
100
67.5

(b)

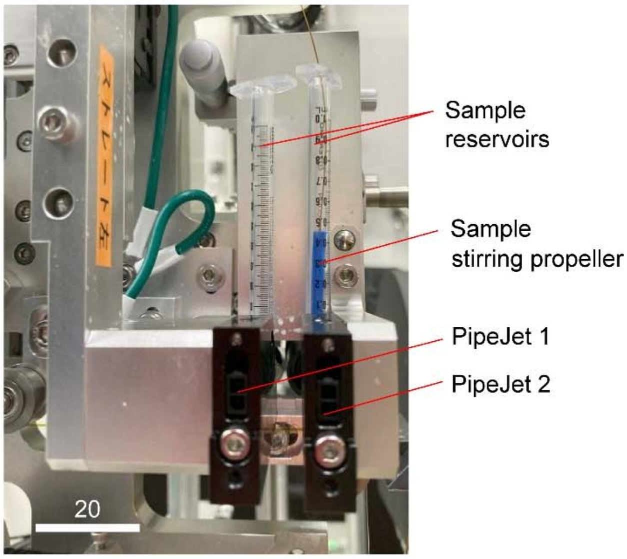
Sample
reservoirs
Sample
stirring propeller
PipeJet 1
PipeJet 2
20

(c)

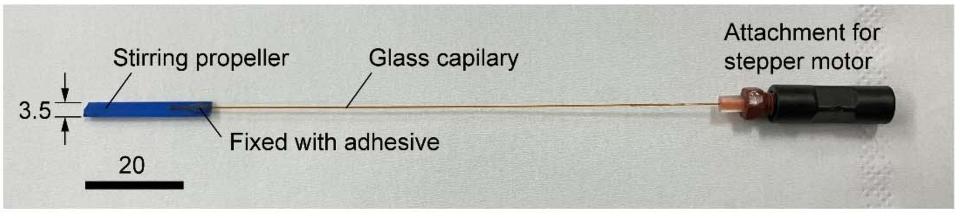
Stirring propeller
Glass capilary
Attachment for
stepper motor
3.5
Fixed with adhesive
20

(d)

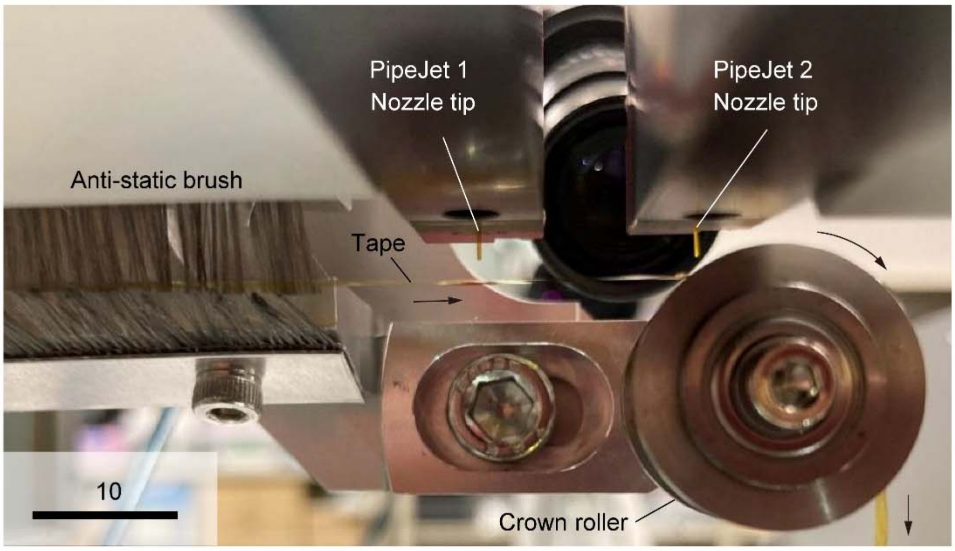
PipeJet 1
Nozzle tip
PipeJet 2
Nozzle tip
Anti-static brush
Tape
10
Crown roller

(e)

Figure S2 Photographs of the piezoelectric injector of PipeJet and ejected droplet queue on the tape surface: (a) 9 nl droplet queue of pure water on the tape surface of the Kapton® film without water-repellent treatment, (b) PipeJet unit mounted with a 1 ml Terumo syringe as the sample reservoir, (c) two PipeJet bodies installed on the ejection head plate with two individual sample reservoirs (PipeJet 1: substrate solution , PipeJet 2: crystal slurry) and a sample stirring propeller inserted in the crystal reservoir, (d) close-up view of the sample stirring propeller, and (e) expanded view of the nozzle tips and their surroundings. The sample stirring propeller in (d) is fabricated by using a commercial wire-reinforced plastic ribbon tie with a width of 3.5 mm and a thickness of 0.5 mm or less called “NEJIRIKKO”. The ribbon can be shaped by hand into forms such as a spiral, and is cut to a sufficient length according to the sample reservoir, dozens of millimetres long, and fixed to a glass capillary of outer diameter 360 µm using adhesive. The attachment part is made to match the stepper motor in use for rotation. The three arrows in (e) indicate the tape travel direction. Units are in millimetres.

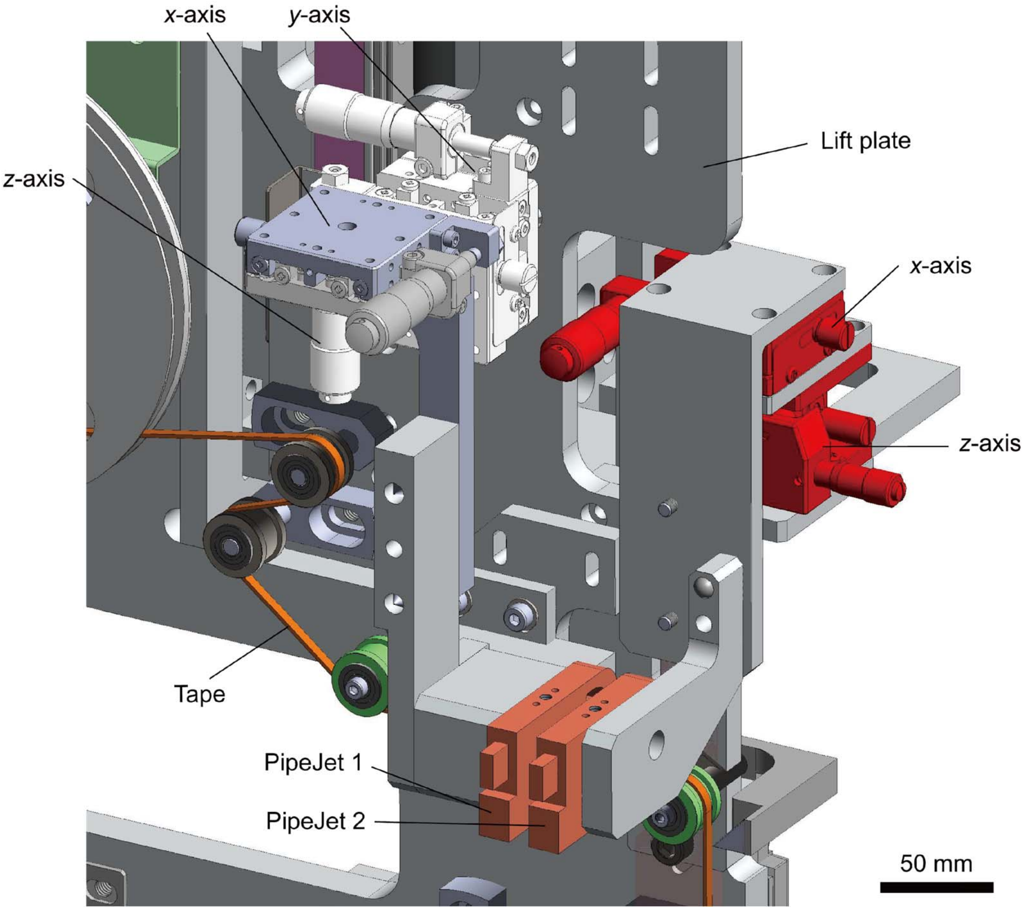

Figure S3 CAD diagram of PipeJet units 1 and 2 mounted on the lift plate with *x*-, *y*-, and *z*-axis micrometer stages and *x*- and *z*-axis micrometer stages, respectively.

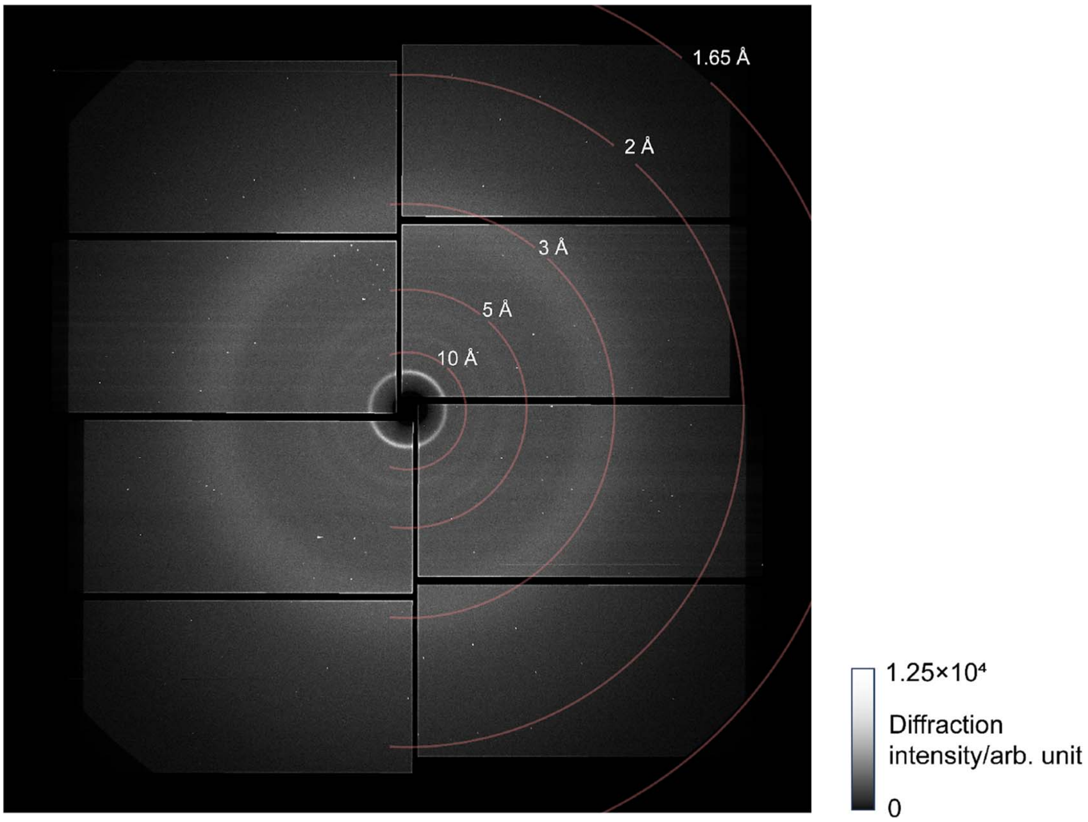
1.65 Å
2 Å
3 Å
5 Å
10 Å
1.25×10⁴
Diffraction intensity/arb. unit
0

(a)

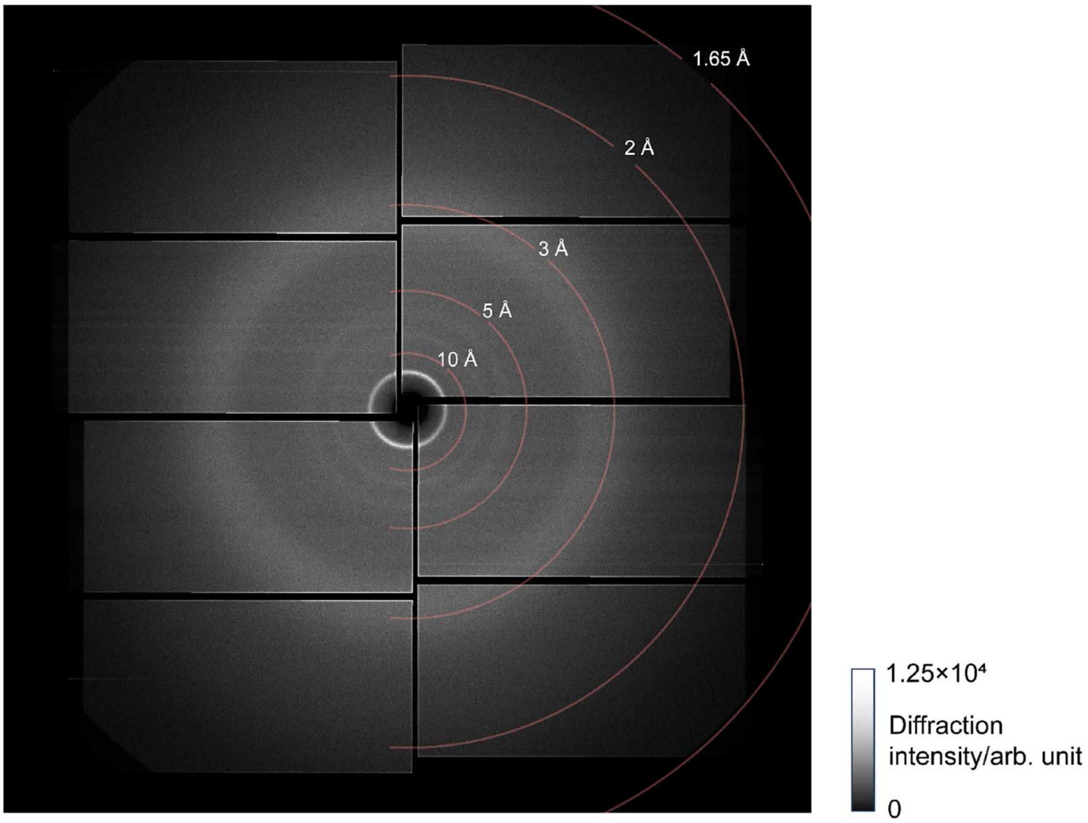
1.65 Å
2 Å
3 Å
5 Å
10 Å
1.25×10⁴
Diffraction intensity/arb. unit
0

(b)

Figure S4 Typical XFEL diffraction patterns recorded by the phase-III MPCCD SWD. (a) The diffraction pattern of a 3–5 µm-HEWL microcrystal without GlcNAc, (b) Scattering patterns from a droplet without crystals on the tape. The XFEL pulse is focused on a 1.5 µm diameter with a photon energy of 10 keV. The pulse energy is around 370 µJ on average. The droplet volume is from 10 to 14 nl, which is estimated to have a height of 90 to 100 µm, respectively. The distance from the sample–XFEL pulse interaction point to the detector surface is 70 mm. The white circles inside of 5 Å are diffraction rings belonging to the 12.5-µm-thick Kapton film.

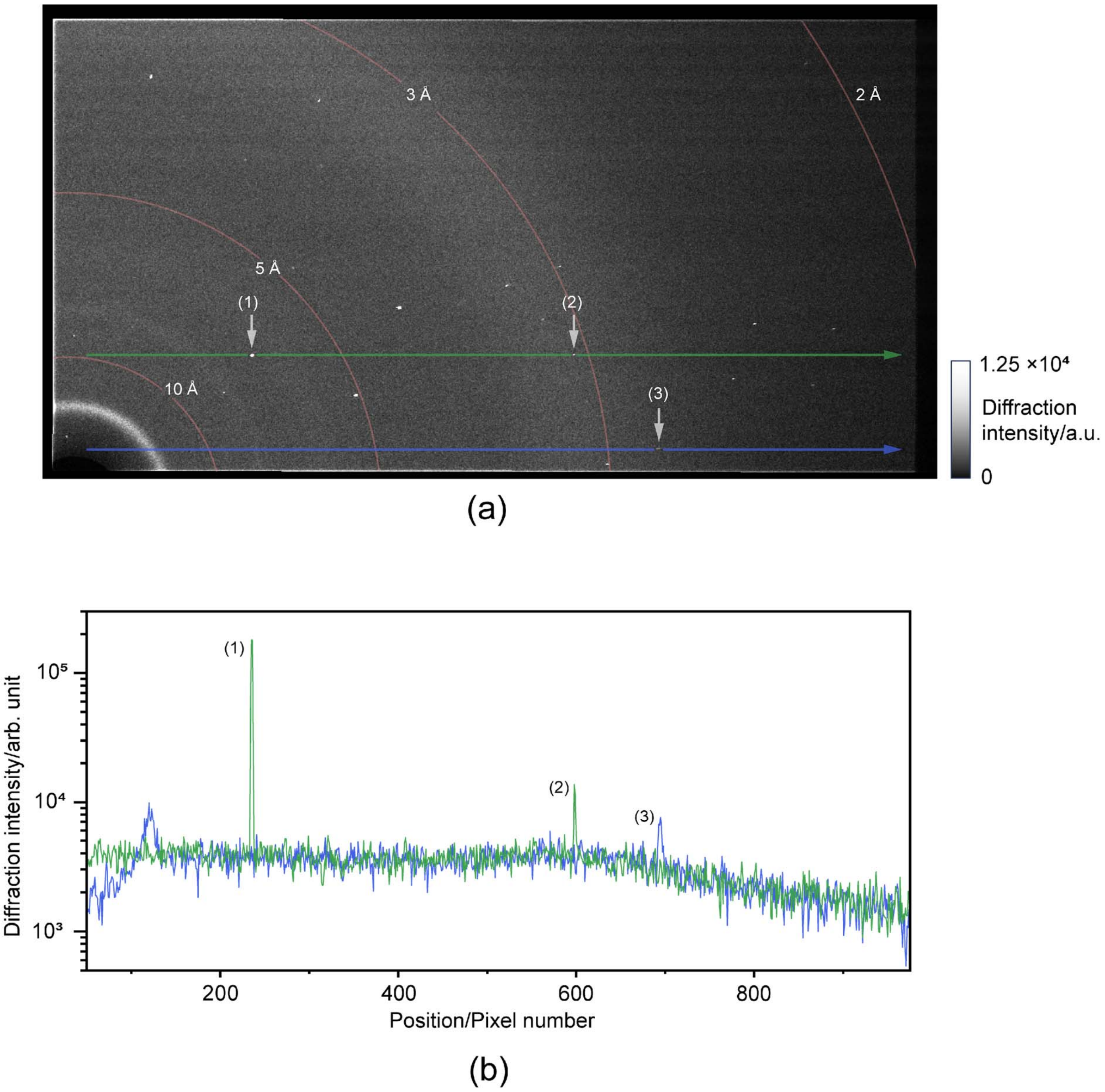

Figure S5 (a) The magnified diffraction patterns of Fig. S4a, the second panel from the top of the right side, (b) the cross-sections of the green line arrow and blue line arrow in S5a.

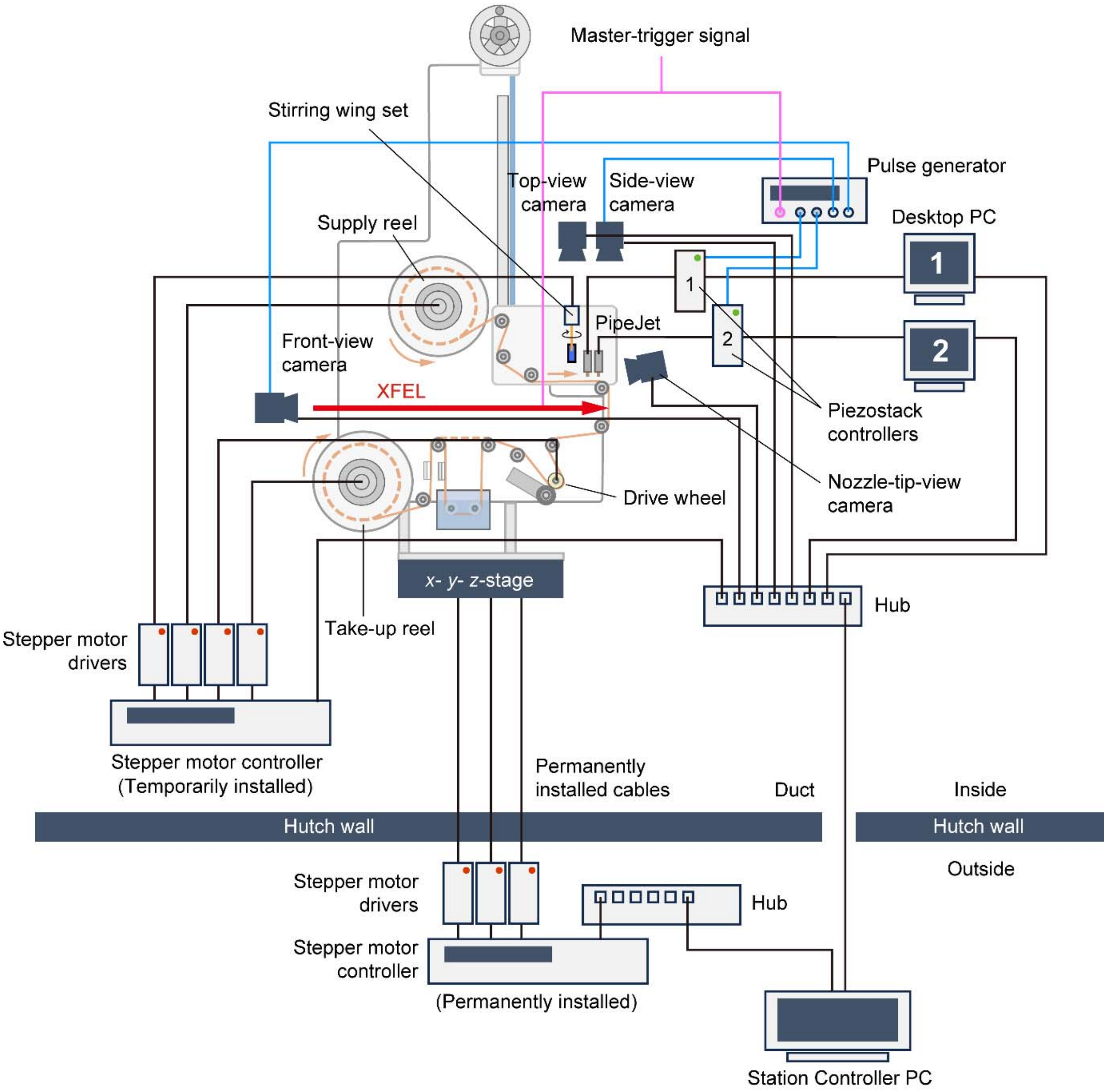

Figure S6 Schematic diagram of CoT inside and outside of the experimental hutch. Two PipeJets are individually connected to two desktop PCs to control the piezostack stroke and stroke velocity separately using Hamilton's bundle software. Both PipeJets and the two cameras, front-view and side-view, are synchronized with XFEL via a pulse generator (DG645) that refers to the timing signal (mainly rising signal) from the master-trigger system of the data acquisition system at SACLA (Joti *et al.*, 2015). For the alignment of the droplet position to the XFEL beam, it is more convenient to adjust the *x*- and *z*-stage in the range of ±1 mm at the tape speed of 30 mm s^{-1} or lower, while shifting the dispensing timing of the PipeJet is more useful at the higher tape speed.

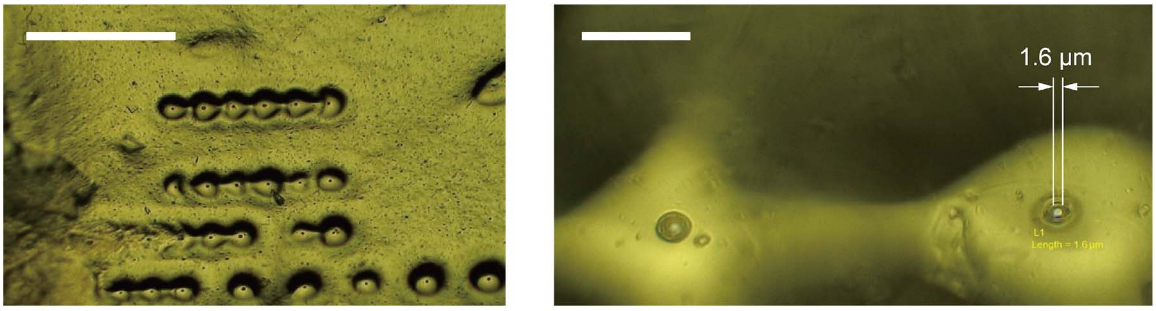

(a) (b)

Figure S7 Photographs of the tape surface of Kapton film after the XFEL irradiation: (a) Traces on the tape surface after XFEL irradiation, and (b) an enlarged view of the traces on the tape surface after XFEL irradiation. A hole punched due to the XFEL has a diameter of 1.6 µm, which is quite close to the XFEL beam size itself. The scale bars indicate (a) 500 µm and (b) 20 µm.

Table S1 Protein concentration estimation based on the number of molecules in HEWL crystals

**Crystal size**	1-µm HEWL crystal	3–5-µm HEWL crystal
Protein molecular weight	14,296 Da $(2.4\times10^{-17}$ mg)	
Protein molecular number per units	8	
Unit cell volume	2.4×10^{-7} µm^3 $(2.4\times10^{-13}$ nl)	
Crystal volume	1 µm^3 $(1\times10^{-6}$ nl)	1.2×10^{2} µm^3 $(1.2\times10^{-4}$ nl)
Unit cell number per crystal	4.2×10^{6}	4.9×10^{8}
Protein molecule number per crystal	3.3×10^{7}	3.9×10^{9}
Protein mass per crystal	7.9×10^{-10} mg	9.3×10^{-8} mg
Crystal density	1.1×10^{9} crystals ml^{-1}	4.8×10^{8} crystals ml^{-1}
Protein concentration	8.7×10^{-1} mg ml^{-1}	4.4×10^{1} mg ml^{-1}

Table S2 Number of crystals and protein mass in a droplet

**Droplet volume**	**10 nl** **(crystal slurry)**	**14 nl** **(crystal slurry)**	**20 nl** **(combined 10 nl droplet of crystal slurry & 10 nl droplet of inhibitor solution)**	**28 nl** **(combined 14 nl droplet of crystal slurry & 14 nl droplet of inhibitor solution)**
Diameter (µm)	520	580	660	740
Height (µm)	91	102	116	130
1 µm crystal numbers in a droplet $(1.1\times10^{9}$ crystals/ml)	11,000	15,400	11,000	15,400
3–5 µm crystal numbers in a droplet $(4.8\times10^{8}$ crystals/ml)	4,800	6,720	4,800	6,720
Protein mass per droplet for 1 µm crystal (µg)	8.7×10^{-3}	1.2×10^{-2}	8.7×10^{-3}	1.2×10^{-2}
Protein mass per droplet for 3–5 µm crystal (µg)	4.4×10^{-1}	6.2×10^{-1}	4.4×10^{-1}	6.2×10^{-1}
Total volume ratio of 1 µm crystal per droplet volume (%)	0.1			
Total volume ratio of 3–5 µm crystal per droplet volume (%)	5.7			
1 µm crystal numbers in the 1.5 µm-diameter XFEL pulse path	0.18	0.20	0.11	0.13
3–5 µm crystal numbers in the 1.5 µm-diameter XFEL pulse path	0.08	0.09	0.05	0.06

Table S3 Sample consumption per dataset

Data set	Elapsed measurement time (second)	Indexed image	Total consumed protein weight per data set (mg) / Consumed protein weight per 10k indexed image (mg)		
			10 nl droplet	14 nl droplet	Average
3–5 µm crystal without inhibitor	1,070	12,213	14.1 / 11.6	19.9 / 16.3	17.0 / 13.9
3–5 µm crystal 226 mM GlcNAc 2 s	890	10,062	11.7 / 11.7	16.6 / 16.5	14.2 / 14.1
3–5 µm crystal 226 mM GlcNAc 5 s	1,958	20,382	25.8 / 12.7	36.4 / 17.9	31.1 / 15.3
3–5 µm crystal 226 mM GlcNAc 9.7 s	1,810	16,607	23.9 / 14.9	33.7 / 21.0	28.8 / 17.9
1 µm crystal without inhibitor	3,354	23,501	0.9 / 0.4	1.2 / 0.5	1.0 / 0.4
1 µm crystal 226 mM GlcNAc 1.3 s	6,632	39,020	1.7 / 0.4	2.4 / 0.6	2.1 / 0.5
1 µm crystal 226 mM GlcNAc 5 s	3,860	16,967	1.1 / 0.6	1.4 / 0.8	1.2 / 0.7
1 µm crystal 226 mM GlcNAc 7.5 s	2,136	11,967	0.6 / 0.5	0.8 / 0.6	0.7 / 0.6
1 µm crystal 226 mM GlcNAc 9.7 s	5,344	17,407	1.4 / 0.8	1.9 / 1.1	1.7 / 1.0
1 µm crystal 452 mM GlcNAc 1.3 s	2,563	15,331	0.7 / 0.4	0.9 / 0.6	0.8 / 0.5
1 µm crystal 452 mM GlcNAc 2.5 s	2,139	12,483	0.6 / 0.5	0.8 / 0.6	0.7 / 0.5
1 µm crystal 452 mM GlcNAc 4 s	3,070	12,723	0.8 / 0.6	1.1 / 0.9	1.0 / 0.8
1 µm crystal 452 mM GlcNAc 5 s	2,351	17,626	0.6 / 0.4	0.9 / 0.5	0.7 / 0.4

Movie S1–S4 Pure water droplets of 9 nl were ejected on the tape at 30 mm s^{-1} (Movie S1), 60 mm s^{-1} (Movie S2), 150 mm s^{-1} (Movie S3), and 300 mm s^{-1} (Movie S4) and recorded by a front camera coaxial with the XFEL beam direction. The camera captured 10 frames per second, with each frame having an exposure time of 750 µs, synchronized with the PipeJet's ejection frequency of 30 Hz. If droplets are located nearly at the same place on the tape, it indicates they are transported stably. In Movie S4, each droplet appears elongated due to high-speed transport at 225 µm per frame. The scale bars indicate 200 µm.

Movie S5 A 5 nl pure water droplet train ejected onto the tape was recorded using a front camera coaxial with the XFEL beam. The camera captured 10 frames per second, with an exposure time of 750 µs per frame. The scale bar indicates 200 µm.

Reference for supporting information

Joti, Y., Kameshima, T., Yamaga, M., Sugimoto, T., Okada, K., Abe, T., Furukawa, Y., Ohata, T., Tanaka, R., Hatsui, T. & Yabashi, M. (2015). *J Synchrotron Radiat* **22**, 571-576.
